# Epigenetic approach in obesity: DNA methylation in a prepubertal population which underwent a lifestyle modification

**DOI:** 10.1186/s13148-020-00935-0

**Published:** 2020-09-23

**Authors:** Cristina Gallardo-Escribano, Verónica Buonaiuto, M. Isabel Ruiz-Moreno, Antonio Vargas-Candela, Alberto Vilches-Perez, Javier Benitez-Porres, Angel Ramon Romance-Garcia, Alejandro Ruiz-Moreno, Ricardo Gomez-Huelgas, M. Rosa Bernal-Lopez

**Affiliations:** 1 Clinical Analysis Department, Regional University Hospital of Malaga, Malaga, Spain; 2 Internal Medicine Department, Instituto de Investigación Biomédica de Malaga (IBIMA), Regional University Hospital of Malaga, Malaga, Spain; 3 Endocrinology and Nutrition Department, Instituto de Investigación Biomédica de Malaga (IBIMA), University Hospital Virgen de la Victoria, Malaga, Spain; 4 Department of Human Physiology, Physical Education and Sports. Faculty of Medicine, University of Malaga, Malaga, Spain; 5 Biodynamic and Body Composition Laboratory. Department of Didactics of Language, Arts, and Sport. Faculty of Education Science, University of Málaga, Malaga, Spain; 6 CIBER Patofisiología de la Obesidad y la Nutrición, Carlos III Health Institute, Madrid, Spain

**Keywords:** Prepubertal population, Metabolically healthy obesity, Methylation, Lipid profile, Inflammatory profile, Lifestyle modification

## Abstract

**Background:**

Metabolically healthy obesity (MHO) is a considerably controversial concept as it is considered a transitory condition towards the development of different pathologies (type 2 diabetes, insulin resistance, or cardiovascular disease). MHO is closely related to lifestyle and environmental factors. Epigenetics has become an essential biological tool to analyze the link between obesity and metabolic status. The aim of this study was to determine whether MHO status is conditioned by the DNA methylation (DNAm) of several genes related to lipid metabolism (lipoprotein lipase, retinoid X receptor alpha, liver X receptor, stearoyl-CoA desaturase, sterol regulatory element binding factor 1), and inflammation (LEP) in peripheral blood mononuclear cells (PBMCs) from 131 prepubertal subjects with MHO phenotype after lifestyle modifications with personalized Mediterranean diet (MedDiet) combined with a physical activity (PA) program.

**Results:**

The DNAm of all studied genes were significantly modified in the population after 12 months of lifestyle modifications (MedDiet and PA). In addition, associations were found between the DNAm studies and BMI, homeostatic model assessment of insulin resistance, monounsaturated fatty acid and polyunsaturated fatty acid, moderate-vigorous PA, fat mass, and adherence to MedDiet.

**Conclusions:**

It was found that DNAm of genes related to lipid metabolism and inflammation are also present in childhood and that this methylation profile can be modified by interventions based on MedDiet and PA.

## Introduction

Obesity is a global health problem that has long-term health repercussions and involves a chronic state of inflammation and increased risk of developing cardiovascular disease, type 2 diabetes, and some types of cancer [1–3]. The World Health Organization (WHO) defines obesity as the abnormal or excessive accumulation of fat that can be harmful to health.

In 2016, the WHO estimated that 41 million children under the age of 5 were overweight or obese (http://www.who.int/dietphysicalactivity/childhood/es/), making obesity the most prevalent nutritional disorder in childhood.

It is known that obesity is a multifactorial entity influenced by environmental (including dietary habits and physical activity (PA)), psychosocial, and neuroendocrine status as well as genetics and epigenetics [4]. The increase in the prevalence of this pathology has led to an increase in the number of studies on its treatment and prevention, including some that study the influence of epigenetics in response to diet and predisposition to weight gain [5]. Childhood and adolescence are critical periods for the development of obesity. Many studies have demonstrated that obesity developed during infancy is associated with a high risk of being overweight in adult life [6]. However, obesity is phenotypically a very heterogeneous pathology and, concretely, includes the metabolically healthy obese (MHO) phenotype. This phenotype describes individuals with obesity who do not meet the criteria of the metabolic syndrome [7]. They may have an epigenetic profile different than that of other phenotypes associated with obesity.

Epigenetics is defined as inheritable and reversible phenomena that affect gene expression without altering the DNA sequence through DNA and histone protein modifications, including DNA methylation (DNAm, the most stable epigenetic modification), covalent histone modifications, chromatin folding, and the regulatory noncoding miRNAs. Epigenetic changes can modify gene transcription by altering the accessibility of gene transcription machinery [8, 9]. These changes are influenced by environmental factors such as nutrition, inflammation, hypoxia, PA, sex, and age, with these epigenetic changes acting as a regulator of the environment-gene interaction and the way the genome responds to lifestyle changes [10, 11].

Obesity is a multifactorial disease involving interactions between an individual’s genetic makeup and an unhealthy lifestyle (unhealthy food intake and sedentarism). It is known that epigenetic modifications affect the expression of different metabolic genes, including lipid metabolism and inflammation genes involved in obesity pathogenesis. Identifying subjects who present with changes in DNAm could help to predict their susceptibility to gain or lose weight, thus helping to prevent obesity via the implementation of new therapeutic approaches [12–15].

A variety of genes have been proposed as candidates for methylation studies. Variations in the methylation profiles of various genes related to the metabolic pathway in obesity are associated with BMI, adiposity, and waist circumference (WC) [16].

The objective of this study was to analyze the methylation profile of genes involved in lipid metabolism after a personalized lifestyle modification based on the Mediterranean diet (MedDiet) and physical activity (PA), during 4-month and 12-month periods, in a prepubertal population of children with metabolically healthy obesity.

## Results

### Anthropometric and clinical changes

The characteristics of the population at baseline, 4 months, and 12 months after the intervention and by gender and for all participants are summarized in Table 1.

**Table 1:** Anthropometric (A) and analytical (B) parameters at baseline, after 4 months, and 12 months of intervention in the total study population and by gender (mean ± SD)

		Baseline (B)	4 months (4 m)	12 months (12 m)	p (B vs 4 m)	p (B vs 12 m)
A						
Body weight (kg)	All	46.2 ± 10.2	46.9 ± 10.5	50.2 ± 11.1	0.6	< 0.001
	Boys	48.2 ± 10.0	48.9 ± 10.5	50.7 ± 11.2	0.7	< 0.001
	Girls	44.0 ± 10.0	44.8 ± 10.1	49.7 ± 11.0	0.7	< 0.001
Height (cm)	All	136.0 ± 9.2	137.9 ± 8.9	141.9 ± 9.1	0.1	< 0.001
	Boys	138.4 ± 7.9	140.3 ± 7.9	143.2 ± 9.0	0.2	< 0.001
	Girls	133.3 ± 9.4	135.2 ± 9.2	140.6 ± 9.1	0.3	< 0.001
BMI (kg/m^2^)	All	24.7 ± 3.5	24.4 ± 3.5	24.7 ± 3.9	0.6	0.1
	Boys	25.0 ± 3.7	24.6 ± 3.7	24.6 ± 4.3	0.6	0.6
	Girls	24.3 ± 3.2	24.2 ± 3.2	24.8 ± 3.6	0.8	0.04
WC (cm)	All	79.8 ± 9.0	80.6 ± 9.7	81.2 ± 9.7	0.5	0.001
	Boys	81.4 ± 9.9	82.4 ± 10.3	82.4 ± 10.5	0.6	0.003
	Girls	78.1 ± 7.7	78.8 ± 8.6	79.9 ± 8.7	0.7	0.1
SBP/DBP (mmHg)	All	107 ± 12/69 ± 11	108 ± 13/70 ± 9	110 ± 12/71 ± 10	0.7/0.3	0.02/0.3
	Boys	108 ± 11/71 ± 11	109 ± 10/70 ± 7	109 ± 10/70 ± 8	0.5/0.7	0.5/0.6
	Girls	105 ± 14/67 ± 10	106 ± 16/71 ± 11	111 ± 13/71 ± 12	0.8/0.1	0.001/0.1
B						
Glucose (mg/dL) NV 70–110	All	81.1 ± 9.1	80.5 ± 9.8	85.4 ± 7.7	0.64	< 0.001
	Boys	81.8 ± 7.7	81,3 ± 10.0	84.8 ± 7.4	0.78	0.03
	Girls	80.2 ± 10.5	79.5 ± 9.6	86.1 ± 8.2	0.71	< 0.001
HbA1c (%) NV 4.0–6.0	All	5.29 ± 0.24	5.29 ± 0.23	5.26 ± 0.24	0.55	< 0.001
	Boys	5.29 ± 0.23	5.27 ± 0.24	5.25 ± 0.23	0.75	0.001
	Girls	5.30 ± 0.24	5.30 ± 0.23	5.28 ± 0.26	0.87	0.05
Insulin (μUI/mL) NV 4.0–16.0	All	21.2 ± 11.5	19.1 ± 10.9	14.6 ± 7.7	0.16	< 0.001
	Boys	20.6 ± 9.7	18.0 ± 8.0	14.0 ± 6.7	0.13	< 0.001
	Girls	22.0 ± 13.2	20.5 ± 12.8	15.3 ± 8.9	0.57	< 0.001
HOMA-IR index NV ≤ 3.4	All	4.3 ± 2.4	3.9 ± 2.6	3.1 ± 1.8	0.19	< 0.001
	Boys	4.2 ± 2.0	3.7 ± 2.1	2.9 ± 1.5	0.18	< 0.001
	Girls	4.5 ± 2.8	4.1 ± 3.0	3.3 ± 2.1	0.55	0.001
Total cholesterol (mg/dL) NV < 200 mg/dL	All	162.1 ± 27.3	161.9 ± 30.0	160.7 ± 26.5	0.9	0.71
	Boys	162.5 ± 29.4	164.8 ± 31.1	160.4 ± 26.6	0.67	0.80
	Girls	161.6 ± 25.0	158.8 ± 28.8	161.0 ± 26.7	0.57	0.33
LDL-c (mg/dL) NV < 130 mg/dL	All	95.2 ± 22.5	95.6 ± 23.4	96.1 ± 23.7	0.93	0.41
	Boys	95.5 ± 24.6	96.9 ± 23.7	96.1 ± 24.9	0.77	0.86
	Girls	95 ± 20.0	93.7 ± 23.2	96.1 ± 22.6	0.79	0.28
HDL-c (mg/dL) NV > 50 mg/dL	All	49.4 ± 11.3	45.9 ± 11.6	48.0 ± 10.7	0.04	0.39
	Boys	49.9 ± 11.4	48.3 ± 11.9	48.5 ± 10.9	0.50	0.51
	Girls	48.7 ± 11.2	42.5 ± 10.5	47.3 ± 10.6	0.01	0.59
TG (mg/dL) NV < 150 mg/dl	All	77.0 (59.0–100.0)	58 (76–99.8)	73 (55–103)	0.75	0.49
	Boys	77.0 (58–100.0)	71 (51–193.8)	72 (52–99)	0.44	0.66
	Girls	77.0 (62.8–99.3)	82.2 (61.0–106.3)	76 (56–107)	0.23	0.60
IL-6 (pg/ml) NV 3.13–12.5	All	0.18 ± 0.16	0.19 ± 0.15	0.98 ± 1.19	0.06	< 0.001
	Boys	0.17 ± 0.16	0.18 ± 0.16	0.88 ± 1.08	0.13	0.001
	Girls	0.20 ± 0.16	0.20 ± 0.15	1.08 ± 1.31	0.30	< 0.001
TNFa (pg/ml) NV < 15.6	All	0.21 ± 0.16	0.19 ± 0.15	0.35 ± 0.38	0.03	< 0.001
	Boys	0.21 ± 0.15	0.18 ± 0.14	0.32 ± 0.34	0.01	< 0.001
	Girls	0.22 ± 0.18	0.19 ± 0.17	0.38 ± 0.43	0.55	< 0.001

(A) *BMI* body mass index, *WC* waist circumference, *SBP* systolic blood pressure, *DBP* diastolic blood pressure

All, *n* = 131 participants (*n* = 70 boys and *n* = 61 girls; *p* = 0.65)

(B) *HbA1c* glycosylated hemoglobin A1c, *HOMA-IR* Homeostatic Model Assessment of Insulin Resistance, *LDL-c* low-density lipoprotein cholesterol, *HDL-c* high-density lipoprotein cholesterol, *TG* triglycerides, *IL-6* interleukin 6, *TNFa* tumor necrosis factor alpha

All, *n* = 131 participants (*n* = 70 boys and *n* = 61 girls; *p* = 0.65)

Regarding anthropometric (Table 1A) variables, participants’ weight and height showed significant differences at 12 months compared to baseline, both in the general population and by sex. After 12 months of intervention, weight and height showed a significant increase with respect to baseline conditions in the total population (+ 4.0 ± 0.9 kg and + 5.9 ± 1.5 cm). When data were analyzed according to sex, significant increases were also observed (boys + 2.5 ± 1.2 kg and + 4.8 ± 1.1 cm; girls + 5.7 ± 1.0 kg and + 7.0 ± 0.3 cm). Of the total population, 91.8% of participants gained weight. In the female group, 46% gained weight compared to 44% in the male group. On the other hand, only 8.2% of the total population lost weight, with boys losing more weight than girls (− 3.0 ± 3.0 kg vs − 0.3 ± 0.2 kg). All participants gained height (100%), with girls gaining slightly more height than boys. No significant changes in BMI were observed in the total population (0.0 ± 0.4 kg/m^2^). When studying the genders separately, BMI decreased slightly in boys (− 0.4 ± 0.2 kg/m^2^) but increased in girls (+ 0.5 ± 0.4 kg/m^2^). Also, WC increased significantly in the total population (1.4 ± 0.7 cm), although this effect was only observed in male sex (1.0 ± 0.6 cm). Finally, SBP increased in the population as a whole (3.0 ± 0.8 mmHg), in this case due to a significant increase in SBP in girls (6.0 ± 0.6 mmHg).

In regard to the analytical parameters (Table 1B), after 12 months of the intervention, the entire population showed a statistically significant increase in fasting blood glucose (within normal range). Insulin levels and homeostatic model assessment of insulin resistance (HOMA-IR) decreased after 12 months of intervention. The lipid profile also underwent several changes, although they were not statistically significant. Both interleukin 6 (IL-6) and tumor necrosis factor alpha (TNFa) levels increased significantly after the intervention.

Analysis of Mediterranean diet adherence in the population showed that it improved by almost 2 points in the total population after 4 and 12 months of intervention (baseline 7.2 ± 1.7; 4 months 8.8 ± 2.0, *p* (B vs 4 m) < 0.0001; 12 months: 8.4 ± 2.0, *p* (B vs 12 m) = 0.01).

### Lifestyle modification

In Table 2, energy intake is summarized. The energy intake significantly decreased after the intervention. In addition, all components of the diet decreased significantly except for the consumption of fiber and vitamin D. Data on energy expenditure reflect significant variations in both levels of sedentarism as well as in light, moderate, and vigorous PA (Table 3) after the intervention. Sedentary time per day increased in the total population in a significant manner (*p* < 0.0001) while moderate and vigorous exercise time both increased as well. Lastly, changes in participants’ body composition (Table 4) showed that lean mass and total mass were significantly increased and total fat decreased in the entire population after 12 months of intervention.

**Table 2:** Energy and food intake at baseline, after 4 months and 12 months of intervention in the total study population and by gender (mean ± SD). All, *n* = 131 participants (*n* = 70 boys and *n* = 61 girls; *p* = 0.65)

		Baseline (B)	4 months (4 m)	12 months (12 m)	p (B vs 4 m)	p (B vs 12 m)
Energy (kcal)	All	2180.2 ± 378.1	2007.5 ± 350.0	1874.3 ± 436.2	0.002	< 0.001
	Boys	2215.0 ± 348.2	2024.3 ± 397.9	1929.4 ± 445.7	0.02	0.001
	Girls	2145.4 ± 405.7	1989.6 ± 296.1	1816.14 ± 424.2	0.04	< 0.001
Total carbohydrates (g/d)	All	228.8 ± 44.1	205.4 ± 44.9	196.8 ± 51.6	< 0.001	0.001
	Boys	2 32.7 ± 44.4	211.0 ± 45.6	204.7 ± 47.3	0.008	0.01
	Girls	224.9 ± 43.9	199.6 ± 44.0	188.6 ± 55.2	0.003	< 0.001
Total protein (g/d)	All	88.7 ± 16.8	82.8 ± 16.7	79.7 ± 19.4	0.07	< 0.001
	Boys	91.3 ± 16.0	85.3 ± 20.42	82.3 ± 20.4	0.06	0.001
	Girls	86.1 ± 17.4	80.2 ± 11.4	77.0 ± 18.1	0.5	0.01
Total fat (g/d)	All	101.3 ± 25.7	94.8 ± 20.6	85.3 ± 23.4	0.5	< 0.001
	Boys	102.7 ± 24.3	93.0 ± 24.7	86.8 ± 25.7	0.3	0.01
	Girls	99.9 ± 27.1	96.7 ± 15.1	83.7 ± 20.8	0.9	0.003
SFA (g/d)	All	32.9 ± 9.7	29.7 ± 8.5	26.6 ± 8.7	0.1	< 0.001
	Boys	33.1 ± 9.3	29.2 ± 9.5	26.8 ± 9.2	0.04	0.004
	Girls	32.7 ± 10.1	30.3 ± 7.8	26.3 ± 8.4	0.7	0.003
MUFA (g/d)	All	44.2 ± 12.0	44.2 ± 9.9	40.9 ± 10.9	0.2	0.05
	Boys	45.2 ± 10.7	43.5 ± 11.6	41.9 ± 12.7	0.9	0.1
	Girls	43.2 ± 13.3	45.0 ± 8.0	39.8 ± 8.7	0.04	0.2
PUFA (g/d)	All	15.6 ± 5.8	13.3 ± 5.1	10.9 ± 4.0	0.03	< 0.001
	Boys	15.7 ± 5.5	13.5 ± 5.5	10.7 ± 3.7	0.2	< 0,001
	Girls	15.6 ± 6.2	13.1 ± 4.6	11.1 ± 4.4	0.07	0.002
Cholesterol (mg/d)	All	373.5 ± 170.5	341.6 ± 87.6	305.6 ± 97.6	0.9	0.001
	Boys	384.1 ± 212.0	345.3 ± 96.7	302.4 ± 89.0	0.7	0.02
	Girls	362.8 ± 115.8	337.6 ± 78.1	308.9 ± 107.1	0.6	0.03
Fiber (g/d)	All	13.3 ± 4.3	13.4 ± 5.0	13.9 ± 6.2	0.5	0.9
	Boys	13.9 ± 4.2	13.4 ± 5.4	14.6 ± 6.4	0.4	0.6
	Girls	12.8 ± 4.4	13.4 ± 4.6	13.2 ± 6.0	0.9	0.6
Vitamin D (μg/d)	All	2.4 ± 2.3	2.6 ± 2.6	2.3 ± 2.5	1.0	0.5
	Boys	2.5 ± 2.4	2.6 ± 2.4	2.5 ± 2.5	0. 4	0.8
	Girls	2.4 ± 2.2	2.6 ± 2.7	2.1 ± 2.5	0.5	0.4

*SFA* saturated fatty acid, *MUFA* monounsaturated fatty acid, *PUFA* polyunsaturated fatty acid

All, *n* = 131 participants (*n* = 70 boys and *n* = 61 girls; *p* = 0.65)

**Table 3:** Energy expenditure measured using a GENEActiv Actigraph GT3X+ accelerometer at baseline, after 4 months and 12 months of intervention in the total study population and by gender (mean ± SD)

		Baseline (B)	4 months (4 m)	12 months (12 m)	*p* (B vs 4 m)	*p* (B vs 12 m)
Sedentarism (min/d)	All	397.0 ± 77.0	365.2 ± 73.6	255.5 ± 85.2	< 0.0001	< 0.0001
	Boys	397.0 ± 68.3	354.2 ± 60.4	243.0 ± 78.4	< 0.0001	< 0.001
	Girls	397.1 ± 85.6	375.7 ± 86.8	268.9 ± 92.1	< 0.0001	< 0.001
Physical activity						
Light (min/d)	All	667.4 ± 72.3	670.2 ± 87.3	789.2 ± 92.0	< 0.0001	< 0.0001
	Boys	663.6 ± 63.2	675.6 ± 93.0	797.5 ± 91.9	< 0.0001	< 0.001
	Girls	671.4 ± 80.9	665.2 ± 82.1	780.4 ± 92.1	< 0.0001	< 0.001
Moderate (min/d)	All	15.3 ± 15.5	35.5 ± 14.8	28.0 ± 17.4	< 0.0001	< 0.0001
	Boys	18.9 ± 18.0	40.5 ± 16.5	31.7 ± 19.9	< 0.0001	0.01
	Girls	11.6 ± 11.5	30.7 ± 11.2	24.0 ± 13.4	< 0.0001	< 0.001
Vigorous (min/d)	All	1.2 ± 3.1	9.1 ± 6.3	7.3 ± 6.4	< 0.0001	< 0.0001
	Boys	1.5 ± 3.9	9.7 ± 7.3	7.8 ± 6.9	< 0.0001	< 0.001
	Girls	1.0 ± 1.8	8.4 ± 5.2	6.7 ± 5.8	< 0.0001	< 0.001

All, *n* = 131 participants (*n* = 70 boys and *n* = 61 girls; *p* = 0.65)

**Table 4:** Body composition as measured by a bone densitometer-DXA at baseline, after 4 months and 12 months of intervention in the total study population and by gender (mean ± SD)

		Baseline (B)	4 months (4 m)	12 months (12 m)	*p* (B vs 4 m)	*p* (B vs 12 m)
Fat mass (kg)	All	18.5 ± 5.3	18.4 ± 5.3	19.2 ± 6.4	0.5	0.01
	Boys	18.6 ± 5.4	18.5 ± 5.6	19.0 ± 6.8	0.6	0.2
	Girls	18.5 ± 5.2	18.4 ± 5.0	19.4 ± 6.0	0.6	0.01
Lean mass (kg)	All	27.0 ± 5.1	28.4 ± 5.5	30.8 ± 5.5	< 0.001	< 0.001
	Boys	28.1 ± 4.7	29.5 ± 4.9	31.7 ± 4.9	< 0.001	< 0.001
	Girls	25.8 ± 5.2	27.3 ± 5.8	29.8 ± 5.9	< 0.001	< 0.001
Total mass (kg)	All	45.5 ± 9.8	46.8 ± 10.1	49.9 ± 11.1	< 0.001	< 0.001
	Boys	46.8 ± 9.7	48.0 ± 10.0	50.6 ± 10.8	0.004	< 0.001
	Girls	44.3 ± 9.8	46.6 ± 10.2	49.2 ± 11.4	< 0.001	< 0.001
Total fat (%)	All	40.3 ± 4.0	39.0 ± 4.4	37.8 ± 4.6	< 0.001	< 0.001
	Boys	39.3 ± 4.1	38.0 ± 4.7	36.8 ± 5.0	0.003	< 0.001
	Girls	41.4 ± 3.7	40.03 ± 3.9	38.9 ± 4.0	< 0.001	< 0.001

All, *n* = 131 participants (*n* = 70 boys and *n* = 61 girls; *p* = 0.65)

### DNAm profiles

Results on the methylation status of the studied genes after 4 and 12 months are summarized in Table 5. Methylation of the lipoprotein lipase (LPL) gene increased in the female group after 4 months (*p* = 0.02) and was maintained at 12 months (*p* = 0.04). Also, methylation of leptin (LEP) gene increased in the female group (*p* = 0.02) after 12 months of intervention. No significant modifications were observed in retinoid X receptor alpha (RXRa) methylation. The methylation level of the liver X receptor (LXR) gene decreased slightly at 4 months in the total population (*p* = 0.03) and in the female group (*p* = 0.04), but returned to baseline levels at 12 months. Regarding stearoyl-CoA desaturase (SCD) gene methylation, a slight decrease was found at 4 months that only continued at 12 months in the male group (*p* = 0.05). For the sterol regulatory element binding factor 1 (SREBF1) gene, methylation levels increased slightly in the total population after 4 months but returned to baseline levels after 12 months. In the male group, methylation levels increased at both times (*p* = 0.03 and *p* = 0.04, respectively) while, in girls, methylation levels decreased at 12 (*p* = 0.03) months.

**Table 5:** Variation in percentage of methylation of genes studied after 4 months and 1 year of a lifestyle intervention

Methylation of gene (%)	Population	Baseline (B)	4 months (4 m)	12 months (12 m)	*p* (B vs 4 m)	*p* (B vs 12 m)
LPL (%)	All	2.5 ± 1.6	2.8 ± 2.0	2.6 ± 1.5	0.4	0.7
	Boys	2.9 ± 1.9	2.8 ± 2.5	2.6 ± 1.5	0.8	0.2
	Girls	2.1 ± 1.1	2.7 ± 1.2	2.6 ± 1.5	0.02	0.04
RXRa (%)	All	18.7 ± 3.3	18.8 ± 2.2	18.9 ± 2.3	1.0	0.7
	Boys	19.4 ± 3.3	18.8 ± 1.7	18.8 ± 2.4	0.2	0.3
	Girls	18.0 ± 3.1	18.8 ± 2.7	19.0 ± 2.2	0.3	0.1
LXR (%)	All	1.0 ± 0.7	0.8 ± 0.7	0.8 ± 0.8	0.03	0.3
	Boys	1.0 ± 0.8	0.8 ± 0.8	0.7 ± 0.7	0.2	0.1
	Girls	0.9 ± 0.6	0.7 ± 0.7	0.9 ± 0.9	0.04	0.8
SCD (%)	All	0.6 ± 1.1	0.4 ± 0.6	0.4 ± 0.6	0.3	0.1
	Boys	0.7 ± 0.9	0.4 ± 0.7	0.3 ± 0.6	0.1	0.05
	Girls	0.5 ± 1.2	0.5 ± 0.6	0.4 ± 0.5	0.8	0.7
SRBEF (%)	All	38.9 ± 9.1	39.9 ± 7.0	38.8 ± 7.2	0.3	1.0
	Boys	37.2 ± 7.2	39.6 ± 4.4	39.8 ± 5.6	0.03	0.04
	Girls	40.8 ± 10.6	40.1 ± 9.1	37.7 ± 8.7	0.6	0.03
LEP (%)	All	35.9 ± 4.4	36.3 ± 3.1	36.8 ± 3.4	0.5	0.1
	Boys	36.0 ± 4.8	36.1 ± 3.1	36.7 ± 2.8	0.9	0.7
	Girls	35.7 ± 4.0	36.5 ± 3.2	36.8 ± 4.0	0.3	0.02

All, *n* = 131 participants (*n* = 70 boys and *n* = 61 girls; *p* = 0.65), *p* (boys vs girls)

### Correlations between DNAm levels and anthropometric parameters, clinical parameters, energy intake and consumption, and body composition

The methylation levels of every gene along with anthropometric variables, analytical parameters, food intake, PA, and body composition all showed statistically significant correlations.

There were associations between anthropometric variables, analytical parameters, energy and food intake, energy expenditure, body composition, and adherence to MedDiet in the MHO prepubertal population. At baseline, the methylation of the LPL gene correlated negatively with abdominal visceral fat (*r* = − 0.24, *p* = 0.03) and positively with adherence to the MedDiet (*r* = 0.22, *p* = 0.04) and moderate (*r* = 0.27, *p* = 0.02) and vigorous (*r* = 0.26, *p* = 0.02) PA. Methylation level of the LXR gene correlated positively with weight (*r* = 0.33, *p* = 0.03), abdominal visceral fat mass (*r* = 0.39, *p* = 0.01), and abdominal total mass (*r* = 0.36, *p* = 0.02) in males. SCD gene methylation correlated positively with energy (*r* = 0.27, *p* = 0.02), total fat (*r* = 0.32, *p* = 0.01), monounsaturated fatty acid (MUFA) (*r* = 0.38, *p* = 0.001), polyunsaturated fatty acid (PUFA) (*r* = 0.25, *p* = 0.04), cholesterol (*r* = 0.27, *p* = 0.02), and vitamin D (*r* = 0.35, *p* = 0.003) intake. SREBF1 gene methylation correlated negatively with moderate (*r* = − 0.42, *p* = 0.001) and vigorous (*r* = 0.34, *p* = 0.002) PA. Methylation of the LEP gene correlated negatively with sedentarism (*r* = − 0.33, *p* = 0.03) and light PA (*r* = − 0.25, *p* = 0.02) and positively with HDL-c levels (*r* = 0.21, *p* = 0.04). In girls, RXRa methylation correlated negatively with energy intake (*r* = − 0.45, *p* = 0.01), fat intake (*r* = − 0.58, *p* < 0.001), and saturated fatty acid (SFA) and MUFA intake (*r* = − 0.45, *p* = 0.01; *r* = − 0.58, *p* < 0.001, respectively) and positively with moderate (*r* = 0.36, *p* = 0.03) and vigorous (*r* = 0.40, *p* = 0.02) PA. In all populations, this gene correlated positively with adherence to the MedDiet (*r* = 0.30, *p* = 0.01).

After 4 months of intervention, the methylation of LPL gene correlated negatively to BMI (*r* = − 0.22, *p* = 0.04), WC (*r* = − 0.23, *p* = 0.03), and abdominal viscera fat (*r* = − 0.23, *p* = 0.04) and positively with adherence to the MedDiet (*r* = 0.33, *p* = 0.04). The methylation level of the LXR gene correlated positively with BMI (*r* = 0.48, *p* = 0.001), WC (*r* = 0.38, *p* = 0.01), abdominal visceral fat mass (*r* = 0.41, *p* = 0.01), abdominal total mass (*r* = 0.39, *p* = 0.01), and fiber intake (*r* = 0.40, *p* = 0.03) in males. The SCD gene methylation correlated negatively with PUFA intake (*r* = − 0.26, *p* = 0.04), Furthermore, in females, it correlated negatively with glucose levels (*r* = − 0.33, *p* = 0.04) and HOMA-IR (*r* = 0.34, *p* = 0.05) and positively with adherence to MedDiet (*r* = 0.24, *p* = 0.03). SREBF1 gene methylation correlated negatively with light PA (*r* = − 0.31, *p* = 0.01) and MUFA intake (*r* = − 0.30, *p* = 0.02) and positively with sedentarism (*r* = 0.28, *p* = 0.02). Methylation of the LEP gene correlated negatively with LDL-c levels (*r* = −0.31, *p* = 0.01). In females, RXRa methylation correlated negatively with SBP (*r* = − 0.38, *p* = 0.02) and abdominal viscera fat (*r* = − 0.24, *p* = 0.03) and positively with HDL-c levels (*r* = 0.49, *p* = 0.01).

Finally, after 12 m of intervention, the methylation of the LPL gene correlated negatively with abdominal visceral fat (*r* = − 0.24, *p* = 0.04) and positively with adherence to the MedDiet (*r* = 0.22, *p* = 0.04) and moderate (*r* = 0.27, *p* = 0.02) and vigorous (*r* = 0.26, *p* = 0.02) PA. In males, RXRa methylation correlated negatively with SBP (*r* = − 0.38, *p* = 0.01) and DBP (*r* = − 0.35, *p* = 0.02) and positively with HDL-c levels (*r* = 0.38, *p* = 0.02) and with adherence to the MedDiet (*r* = 0.38, *p* = 0.02). In males, methylation levels of the LXR gene correlated negatively with cholesterol intake (*r* = − 0.42, *p* = 0.01). SCD gene methylation correlated positively with insulin levels (*r* = 0.33, *p* = 0.005) and HOMA-IR (*r* = 0.27, *p* = 0.02). Methylation of the LEP gene correlated negatively with light (*r* = − 0.38, *p* = 0.02) and vigorous (*r* = − 0.36, *p* = 0.07) PA and positively with insulin levels (*r* = 0.36, *p* = 0.03) in females.

To study the strength of the association observed in the correlation analyses, we performed linear regression analyses. We observed that the nutrition, PA, and analytical parameters could explain DNAm levels of our target genes (Tables 6), a regression that was corrected for BMI. Baseline DNAm levels of the LPL gene correlated negatively with energy intake (*p* = 0.03) and hsPCR levels (*p* = 0.01) and positively with carbohydrate intake (*p* = 0.02), protein (*p* = 0.04), MUFA (*p* = 0.01), PUFA (*p* = 0.02), MedDiet adherence (*p* = 0.03), and resistin levels (*p* = 0.03). In addition, DNAm levels of the LEP gene at baseline correlated negatively with BMI (*p* = 0.02) and vigorous PA (*p* = 0.05) and positively with moderate PA (*p* = 0.05). After 12 months of intervention, this gene correlated negatively with light PA (*p* = 0.02). On the other hand, DNAm levels of the SREBF gene correlated negatively at baseline with moderate PA (*p* = 0.01) and positively with glucose levels (*p* = 0.002), HOMA-IR (*p* = 0.01), and hsPCR levels (*p* = 0.01). DNAm levels of the RXRa gene correlated negatively with hsPCR levels (*p* = 0.03) at baseline and negatively with both light and vigorous PA (*p* = 0.01, *p* = 0.03, respectively) after 12 months. DNAm levels of the LXR gene correlated negatively with glucose levels (*p* = 0.02) and positively with HOMA-IR index (*p* = 0.03) at 12 months. Lastly, DNAm levels of the SCD gene correlated negatively with HOMA-IR index (*p* = 0.03), high-density lipoprotein cholesterol (HDL-c) (*p* = 0.01), resistin (*p* = 0.01), and IL-6 (*p* = 0.001) levels and positively with insulin (*p* = 0.01) and TNFa (*p* = 0.02) levels.

**Table 6:** Linear regression analysis with DNA methylation (DNAm) as the dependent variable and nutrition components, physical activity, or analytical parameters as independent variables and corrected for BMI

Nutrition								
DNAm	BMI		Energy intake		Total carbohydrates		Total protein	
Baseline	*ß*	*p*	*ß*	*p*	*ß*	*p*	*ß*	*p*
LPL (*R* = 0.46; *R*^2^ = 0.21)			− 0.03 (0.06 to − 0.04)	0.03	0.14 (0.02–0.253)	0.02	0.12 (0.01–0.23)	0.04
LEP (*R* = 0.34; *R*^2^ = 0.11)	− 0.50 (− 0.92 to − 0.08)	0.02						
	MUFA		PUFA		Adherence to the MedDiet			
	*ß*	*p*	*ß*	*p*	*ß*	*p*		
LPL (*R* = 0.46; *R*^2^ = 0.21)	0.35 (0.09–0.61)	0.01	0.29 (0.05–0.53)	0.02	0.28 (0.02–0.53)	0.03		
Physical activity								
DNAm	Light activity		Moderate activity		Vigorous activity			
Baseline	*ß*	*p*	*ß*	*p*	*ß*		*p*	
SREBF (*R* = 0.44; *R*^2^ = 0.19)			− 0.43 (− 0.74 to − 0.11)	0.01				
LEP (*R* = 0.35; *R*^2^ = 0.13)			0.13 (0.002–0.26)	0.05	− 0.62 (− 1.23 to − 0.001)		0.05	
DNAm	Light activity		Moderate activity		Vigorous activity			
12 m	*ß*	*p*	*ß*	*p*	*ß*		*p*	
RXRa (*R* = 0.39; *R*^2^ = 0.15)	− 0.01 (− 0.02 to − 0.003)	0.01			− 0.11 (− 0.21 to − 0.01)		0.03	
LEP (*R* = 0.35; *R*^2^ = 0.13)	− 0.02 (− 0.03 to − 0.003)	0.02						
Analytical parameters								
DNAm	Glc		HOMA-IR		hsPCR		Resistin	
Baseline	*ß*	*p*	*ß*	*p*	*ß*	*p*	*ß*	*p*
LPL (*R* = 0.47; *R*^2^ = 0.23)					− 0.22 (− 0.39 to − 0.05)	0.01	0.20 (0.02–0.38)	0.03
RXR (*R* = 0.52; *R*^2^ = 0.27)					− 0.48 (− 0.91 to − 0.06)	0.03		
SREBF (*R* = 0.51; *R*^2^ = 0.26)	0.71 (0.27–1.16)	0.002	2.16 (0.65–3.66)	0.01	2.16 (0.65–3.66)	0.01		
DNAm	Glc		Insulin		HOMA-IR		cHDL	
12 m	*ß*	*p*	*ß*	*p*	*ß*	*p*	*ß*	*p*
LXR (*R* = 0.52; *R*^2^ = 0.27)	− 0.08 (− 0.15 to − 0.01)	0.02			1.90 (0.20–3.60)	0.03		
SCD (*R* = 0.75; *R*^2^ = 0.57)			0.25 (0.05-0.45)	0.01	− 1.01 (− 1.96 to − 0.07)	0.03	− 0.02 (− 0.03 to − 0.004)	0.01
	Resistin		IL-6		TNF-a			
	*ß*	*p*	*ß*	*p*	*ß*	*p*		
SCD (*R* = 0.75; *R*^2^ = 0.57)	− 0.04 (− 0.07 to − 0.01)	0.01	− 0.03 (− 0.04 to − 0.01)	0.001	0.05 (0.01–0.1)	0.02		

## Discussion

Personalized lifestyle modification is a great tool for analyzing the pattern in DNA modification from peripheral blood mononuclear cells (PBMCs). Epigenetic marks can change due to metabolic status, dietary intake or PA [17]. In this work, we studied these variations in DNAm in prepubertal population with obesity that was metabolically healthy. After 12 months of intervention, our population showed an improvement in insulin resistance and hyperinsulinemia compared to baseline conditions, components that play a fundamental role in the development of the obesity-related cardiometabolic risk factors [18]. This improvement in clinical parameters was produced as a result of a decrease in intake of different nutrients and total fat and an increase in moderate-vigorous PA. Both lean mass and total mass decreased after 12 months of intervention in the entire population.

Epigenetic marks are used in studies as predictors of the response to weight loss programs. As in our intervention, the DNA has different methylation patterns at the beginning of the study in comparison to the end of follow-up, data supported by Moleres et al. [19]. Some studies have also proposed epigenetics as a predictor of the predisposition towards developing obesity in both childhood and adulthood [16]. Herrera et al. [20] demonstrated the importance of an obesogenic environment. Early environmental influences induce epigenetic variation, which permanently affects metabolism and risk of chronic disease. Evidence suggests that the establishment of the epigenome can be affected by environmental factors during critical developmental periods [21]. There are potential interactions between lifestyle and epigenetic mechanisms that mediate the expression of genes associated with increased BMI and adiposity, as is suggested by the effect of diet on the methylation of genes involved in lipid metabolism and inflammation such as leptin [22]. On the other hand, dietary intake of methyl groups (choline, methionine, genistein, and folate) during critical periods of developmental alters DNA promoter and histone methylation, thereby resulting in lifelong changes in gene expression and alteration of the epigenome that inclines the individual towards obesity in adulthood [23]. Some cross-sectional studies have reported a significant association between obesity or adiposity status, and DNA methylation [24]. These studies demonstrate that some regions of different genes studied were hypomethylated in obese children and located in the gene body region, and revealed a unique cluster of obese individuals that was differentiated from the normal-weight children [25]. Some of these genes are implicated in lipid and glucose metabolism, differential body size, and body composition in children [26].

The genes studied are involved in lipid metabolism and inflammation. The implication of these genes in the obesity process is still unknown. In this context, we observed that the methylation pattern is modified after different periods of follow-up (4 and 12 months) with lifestyle modification (MedDiet and PA) which, according to other studies, showed the relationship between obesity and epigenetic marks. In our study, BMI, an indicator of body adiposity, influenced DNAm. After 12 months of intervention, we verified that the DNAm is located in genes involved in lipid metabolism and inflammatory pathways, results in concordance with Wahl et al. [27]. As far as is known, LPL mediates the used of blood triglycerides. It is a hydrolytic enzyme located in the capillary endothelium that catalyzes the cleavage of triglycerides into fatty acids. Therefore, LPL activity is critical for plasma triglyceride (TG) clearance and tissue uptake of fatty acids [28]. We found a significant correlation between the LPL DNAm levels and adherence to the MedDiet, moderate and vigorous PA, BMI, WC, and abdominal visceral fat at all points of follow-up, in line with previous results [29, 30]. LPL is expressed in different tissues, including adipose tissue and foam cells, and its expression is regulated by LXR/RXR heterodimers. LPL activity is closely linked with RXR [31]. RXRa is a member of the steroid/thyroid hormone superfamily of nuclear receptors that function as transcription factors binding to promoter regions of genes. These receptors exert their action by binding, as homodimers or as heterodimers, to specific sequences in the promoters of the target genes and the regulation of their transcription. In our population, we found DNAm of RXRa had a significant correlation with energy, fat, SFA and MUFA intake, moderate and vigorous PA, and adherence to the MedDiet at baseline. During the follow-up, the DNAm of RXRa showed a significant correlation with HDL-c levels and fat mass at the end of intervention [32]. It is known that RXR heterodimerizes with subfamily 1 nuclear receptors, including LXR [33]. LXRs are members of the nuclear hormone receptor superfamily of ligand-activated transcription factors that regulate cholesterol and lipid metabolism. Their regulation of gene expression in response to changes in intracellular cholesterol levels link lipid metabolism, inflammation, and immune cell function [34].

LXR can be activated by increased intracellular cholesterol concentrations [35]. According to these data, in our MHO prepubertal population, the DNAm of LXR significantly correlated with weight, BMI, WC, cholesterol and fiber intake, and body composition during the intervention’s follow-up. In addition, we found a significant correlation with the DNAm of SCD. SCD is a key enzyme in the conversion of polyunsaturated fatty acids (PUFAs) to monounsaturated fatty acids (MUFAs), and it has been shown to be overexpressed in adipose tissue in obesity. The DNAm of SCD correlated in our population with energy, total fat, PUFA, MUFA, and cholesterol and vitamin D intake at baseline, in accordance with other data [29]. Recent studies suggest that decreased functional SCD promotes lipid oxidation over storage, decreased lipogenesis, increased beta-oxidation, and increased glucose utilization [36]. In this sense, during the follow-up on our participants, the DNAm of SCD correlated with glycemic metabolism parameters (glucose, insulin levels, and HOMA-IR) [37] and adherence to the MedDiet.

Within lipid metabolism, SRE sites are fundamental in the regulation of LPL gene expression by sterols. On the other hand, sterols contribute to the control of triglyceride metabolism via binding of SREBF1 to the LPL regulatory sequences [38]. SREBPs are loop-helix leucine zipper transcription factors that regulate the synthesis and cellular uptake of cholesterol and fatty acids [39]. According to the relation of this gene to fat metabolism, we found correlations with the DNAm of LPL, SCD, and RXRa. In addition, DNAm of SREBF1 significantly correlates with PA and MUFA intake.

Finally, leptin is a hormone secreted by adipocytes whose function is to inhibit intake and stimulate energy expenditure, thus allowing for body weight maintenance. Leptin resistance is related to diet-induced obesity (high-fat diets), which is the main cause of obesity in humans. The decrease in leptin sensitivity leads to deregulation of satiety, which increases intake and affects nutrient absorption, metabolism, insulin sensitivity, and energy balance [40]. In our population, DNAm of leptin correlated with sedentarism and HDL-c levels at baseline. It is known that leptin can regulate cholesterol-ester metabolism and the atherosclerotic process associated with obesity [41]. A significant association has also been found between the DNAm of leptin and insulin and LDL-c levels as well as vigorous PA at the end of follow-up. In accordance with these data, some studies about dietary intervention identify DNAm as biomarkers of a weight loss response [42]. These genomic loci are located in proximity to obesity-associated genes such as LEP. These epigenetic signatures may potentially be used in precision medicine as baseline biomarkers to predict the effectiveness of a weight loss intervention [5].

## Limitations

The limitations of this study include the low weight loss and lack of big changes in the BMI of our participants. However, the period of follow-up of the lifestyle modification of our prepubertal population, which took place over a year, is a strength of this study, since it provides an evaluation of the impact of nutrition and PA over the epigenetic modifications of different genes involved in the inflammation process and lipid metabolism. In addition, MedDiet adherence questionnaire used in this study is validated for Mediterranean populations, but not specifically for a prepubertal population. Another limitation is that there was no randomization or control group (normal-weight subjects) to safely attribute changes, though the results and the longitudinal design are strengths.

## Conclusion

This study shows the results of the DNAm of specific genes related to lipid metabolism and inflammation in metabolically healthy obesity and in a prepubertal population. The DNAm of the genes studied are closely related to different anthropometric and analytical parameters, components of food intake, type of PA, and body composition as well as between those genes. In addition, these results also suggest that the influence of methylation profile is present even in childhood and that this methylation profile can be modified by interventions based on MedDiet and PA.

In terms of public health, a healthy lifestyle in the entire population from childhood to old age is essential so that the epigenetic modifications that occur in human DNA do not lead to the development of different molecular process and pathologies associated with obesity, such as insulin resistance, dyslipidemia, inflammatory processes, hypertension, alterations of the vascular structure, and cardiovascular diseases.

## Material and methods

### Study population

In this study, a prepubescent population aged 4–9 years, of both sexes, was included. Inclusion criteria were boys (from 4 years to testicular volume < 3 ml) and girls (from 4 years to Tanner S2, breast bud elevation) with obesity (≥ 95 percentile) [43]. Participants were metabolically healthy, meaning they presented ≤ 1 of the following criteria: WC and blood pressure ≥ 90 percentile, triglycerides > 150 mg/dl, HDL-c < 40 mg/dl, or impaired fasting glucose [44]. Participants who met more than one criterion of metabolic syndrome, did not meet the age criterion at the start of the study, were diabetic, or had any metabolic pathology were excluded from the study.

The recruitment period was from November 2016 to May 2017 and took place in different schools in the city of Málaga (Andalusia, Spain). Recruitment was carried out via visits to pre-school and elementary school classrooms. Once the possible participants were selected, families were contacted to inform them about the study and invite them to participate. The selected subjects and their parents/guardians were summoned to the Civil Hospital of Malaga in order to inform them about the design and objectives of the study as well as to request their written informed consent for the voluntary participation of their children in the study.

### Study design

The subjects who wished to participate in the study had an initial visit with a nurse. Weight, height, BMI, WC, hip circumference, waist-hip ratio, and blood pressure measurements were taken. Next, blood samples were taken to analyze analytical parameters (triglyceride levels, HDL-c, total cholesterol, low-density lipoprotein cholesterol (LDL-c), glycosylated hemoglobin A1c (HbA1c) (%), creatinine, uric acid, microalbuminuria, insulin, HOMA-IR, and glucose after 12 h of fasting) to confirm that they met the inclusion criteria as well as to determine baseline levels before the intervention. The samples were tested in the Clinical Analysis Laboratory of the Regional Hospital of Malaga. Lastly, lifestyle questionnaires (nutritional questionnaires on the frequency of food consumption, a PA questionnaire, and questionnaires on adherence to the Mediterranean diet) were completed.

Once the parents/guardians granted permission for the inclusion of their child in the study and it was verified that the child met the inclusion criteria, they had an interview with a nutritionist at the beginning of the study. The nutritionist informed the parents/guardians and the children about the characteristics and recommendations of the Mediterranean diet and the healthy lifestyle they should follow during the study period. The participants and their parents/guardians participated in an interview with the nutritionist in which they completed lifestyle questionnaires (nutritional questionnaires about the frequency of food consumption, PA questionnaires, and questionnaires about adherence to the Mediterranean diet) in order to know their lifestyle prior to the intervention. Dietary records completed for three non-consecutive days and a food frequency questionnaire for the previous year (number of times/day, number of days/week, number of days/14 days, number of days/month, rarely, or never) were completed at the baseline visit and 4 and 12 months after the intervention [45] (https://www.healthychildren.org/Spanish/healthy-living/nutrition/Paginas/Energy-In-Recommended-Food-Drink-Amounts-for-Children.aspx). A food frequency questionnaire divided into nine sections showing food groups was used. These sections are diary, eggs, meat and fish, fruits, vegetables, legumes and cereals, oils and fats, pastries, miscellaneous, and beverages. The FFQ includes 9 possible responses for frequency of consumption ranging from never/rarely to > 6 servings/day. In addition, a self-reported record of food questionnaire was used for three consecutive days, 2 days of daily, and another day of the weekend.

The Mediterranean diet recommended to the study subjects included extra virgin olive oil and nuts. The recommended calorie intake was 1500 kcal/day (https://www.healthychildren.org/Spanish/healthy-living/nutrition/Paginas/Energy-In-Recommended-Food-Drink-Amounts-for-Children.aspx) with the following macronutrient distribution [46]: 30% fats (5–8% saturated fatty acids, 15–18% monounsaturated fatty acids, 5–8% polyunsaturated fatty acids, and less than 300 mg of cholesterol per day), 50% carbohydrates (less than 10% simple sugars, 40% complex sugars, and low glycemic index), and 20% protein. Adherence to the Mediterranean diet was determined according to the instructions of Trichopoulou et al. [47].

They were also informed that they should perform regular PA with two trained supervisors with a degree in the physical activity and sports sciences field. International PA guidelines recommend that children exercise daily (http://www.health.gov/paguidelines/chapterthree). To do so, participants were given access to sports programs. We offered the possibility of attending an exercise program daily from Monday to Friday (5 sessions/week for 120 min/session). Participants were required to attend a minimum of 3 sessions per week. Attendance was recorded at the beginning of each PA session. The sessions consisted of aerobic exercises, resistance training, and exercises to improve flexibility and balance. Sessions were held for the duration of the study. In addition, participants kept a PA record using a GENEActiv Actigraph GT3X+ accelerometer to measure energy expenditure. It was used for one full week at the baseline visit and again at 4 and 12 months.

In addition, parents/guardians were provided with the telephone number of those in charge of the study so that they could ask questions about nutrition and PA that may have arisen before the next visit.

All patients participating in the study gave their informed consent, and protocols were approved by the institutional ethics committee (Comité de Ética de la Investigación Provincial de Málaga, belonging to the Andalusian Health Service).

### Epigenetic study

For methylation analysis, PBMCs were used to extract genomic DNA using TRIzol™ Reagent. The epigenetic profiling of genomic DNA was analyzed in 131 MHOCh using the methylation assays (Qiagen). Bisulfite conversion of DNA samples was performed using the EZ DNAm kit (Zymo Research, Orange, CA, USA) on 500 ng of DNA. A PCR of methylated DNA was carried out to amplify the genes of interest using PyroMark PCR Kit (Qiagen) and Primers PyroMark CpG Assays (Qiagen). Pyrosequencing was carried out using PyroMark Gold Q96 and primers PyroMark CpG Assay (Qiagen). The proportion of methylation (%) for each subject at each CpG site was computed by first subtracting the background signal intensity of negative controls from both the methylated and unmethylated signals then dividing the ratio of the methylated signal intensity by the sum of both methylated and unmethylated signals.

Table 7 shows 6 amplicons for the validated CpGs. These gene candidates are lipoprotein lipase (LPL), retinoid X receptor alpha (RXRa), liver X receptor (LXR), stearoyl-CoA desaturase (SCD), sterol regulatory element binding protein (SREBF1), and leptin (LEP).

**Table 7:** Gene characteristics of differentially methylated CpGs

Gene symbol	Gene name	Ref. sequence	Assay ID	Length (bp)
Lipid metabolism				
LPL	Lipoprotein lipase	PM00037401	Hs_LPL_01_PM PyroMark CpG assay	169
RXRa	Retinoid X receptor alpha	PM00144431	Hs_RXRA_01_PM PyroMark CpG assay	86
LXR**/**NR1H2	Liver X receptor	PM00190260	Hs_NR1H2_01_PM PyroMark CpG assay	108
SCD	Stearoyl-CoA desaturase	PM00042196	Hs_SCD_03_PM PyroMark CpG assay	159
SREBF1	Sterol regulatory element binding factor 1	PM00178087	Hs_SREBF1_01_PM PyroMark CpG assay	136
Lipid metabolism and inflammation				
LEP	Leptin	PM00129724	Hs_LEP_01_PM PyroMark CpG assay	98

### Statistical analysis

Quantitative variables were expressed as mean ± standard deviation (SD), and qualitative variables were expressed as percentages. Student’s *t* test was used to compare quantitative variables whereas the chi-square test was used for qualitative variables in order to contrast variables measured within each group at different time periods. Bivariate correlations were determined using the Pearson correlation coefficient analysis. To calculate the sample size (calculated using SISA, Simple Interactive Statistical Analysis), we relied on other clinical studies that have shown the metabolic benefits of weight loss in the pediatric population with obesity [48]. Assuming a 95% confidence level (*α* error of 0.5%), a statistical power of 80%, and a 5% loss rate, a sample of 110 MHO subjects was required. To account for non-participation and possible loss to follow-up, recruitment of at least 130 participants was planned. Statistical analysis was performed using SPSS for Windows, version 22.0 (IBM Corporation INC. Somers, NY, USA).

## Abbreviations

WHO: World Health Organization
MHO: Metabolically healthy obese
DNAm: Deoxyribonucleic acid methylation
miRNAs: Micro ribonucleic acid
MedDiet: Mediterranean diet
PA: Physical activity
BMI: Body mass index
WC: Waist circumference
SBP/DBP: Systolic blood pressure/diastolic blood pressure
HbA1c: Glycosylated hemoglobin A1c
HOMA-IR: Homeostatic model assessment of insulin resistance
LDL-c: Low-density lipoprotein cholesterol
HDL-c: High-density lipoprotein cholesterol
TG: Triglycerides
IL-6: Interleukin 6
TNFa: Tumor necrosis factor alpha
LPL: Lipoprotein lipase
RXRa: Retinoid X receptor alpha
LXR: Liver X receptor
SCD: Stearoyl-CoA desaturase
SREBF1: Sterol regulatory element binding factor 1
LEP: Leptin
SFA: Saturated fatty acid
MUFA: Monounsaturated fatty acid
PUFA: Polyunsaturated fatty acid
PBMCs: Peripheral blood mononuclear cells
